# Neural markers of working memory and emotional regulation are predictors of future depression severity in at risk young adults: a replication study

**DOI:** 10.1038/s41380-026-03771-5

**Published:** 2026-07-31

**Authors:** Yvette Afriyie-Agyemang, Michele A. Bertocci, Satish Iyengar, Richelle S. Stiffler, Lisa K. Bonar, Haris A. Aslam, Simona Graur, Genna Bebko, Osasumwen Benjamin, Yiming Wang, Henry W. Chase, Mary L. Phillips

**Affiliations:** 1 Department of Psychiatry, University of Pittsburgh School of Medicine, Pittsburgh, PA USA; 2 Department of Statistics, University of Pittsburgh School of Arts and Sciences, Pittsburgh, PA USA

**Keywords:** Neuroscience, Predictive markers, Psychology

## Abstract

Objective neural markers that reflect the underlying pathophysiological mechanisms of affective disorders are needed to facilitate early identification of individuals most at risk of future affective disorders and ultimately provide neural targets to guide therapeutic interventions. Using an emotional n-back paradigm designed to examine working memory (WM) and emotional regulation (ER) capacity, we previously showed that WM-related elevated left dlPFC activity (a key node of the central executive network (CEN)) and elevated right precuneus activity (a key node of the default mode network (DMN)), as well as ER-related elevated left dlPFC activity were positively associated with future depression severity in young adults at risk for affective disorders. We now aimed to replicate and extend these previous longitudinal findings by examining relationships among right precuneus activity and left dlPFC activity during WM and ER tasks and future depression severity in a new independent young adult sample (n = 77: 50 female, age = 24.68), and a larger combined sample (n = 121: 83 female, age = 23.81) comprising the original and new samples. The Hamilton Rating Scale for Depression (HAM-D) and Young Mania Rating Scale (YMRS) were measured at 12 months post scan to assess future depression and mania/hypomania severity respectively. In the new sample, we showed patterns of left dlPFC activity and right precuneus activity during WM, and left dlPFC activity during ER that were consistent with the original sample. In both the new and combined samples, future depression severity was robustly predicted by WM-related left dlPFC activity and right precuneus activity, and ER-related left dlPFC activity (all ps < 0.05 qFDR). These findings were specific to future depression severity. The effect sizes (pseudo R-squared values) for the full models including all IVs in the new and combined samples ranged from approximately 25–43%, with left dlPFC and right precuneus activity during WM explaining 15.59% of variance in future depression severity in the new sample; and left dlPFC activity during ER explaining 14.63% of variance in future depression severity in the combined sample. These replicated, longitudinal findings provide candidate neural markers to guide risk identification and targeting of new interventions for individuals with and those at risk for future affective disorders.

## Introduction

Affective disorders, including major depressive disorder (MDD) are chronic, disabling affective illnesses that affect millions of individuals globally [1]. A leading cause of global disability [2], affected individuals exhibit profound mood, cognitive, and behavioral dysregulation [3], resulting in impairments across psychosocial, occupational, and functional domains [4, 5]. Objective neural markers that reflect the underlying pathophysiological mechanisms of MDD are therefore needed to facilitate early identification of individuals most at risk of future MDD and ultimately provide neural targets to guide therapeutic interventions. Furthermore, neural markers of mechanisms specific to risk for depression rather than mechanisms generalizable to risk for mania/hypomania, a core feature of Bipolar Disorder (BD), can be used to guide targeting of antidepressant interventions without concerns about increasing mania/hypomania risk in individuals with vulnerability to both depression and mania/hypomania, i.e., BD [6].

Emotional dysregulation, characterized by frequent use of maladaptive emotional regulation (ER) strategies, is a core feature of affective disorders, and is associated with poorer functional outcomes [7–12]. Effective ER relies on working memory (WM), the process of actively maintaining and manipulating information [13], to support flexible control of emotional responses [12, 14]. More specifically, the ability to redirect attention away from emotional distractors during WM is considered a subprocess of ER [9, 15] and is frequently used to assess ER capacity in young adults at risk for affective disorders [15–21]. Furthermore, deficits in both WM and ER have been linked to current and future affective psychopathology [22–24], particularly depression [11, 25, 26] making them key processes through which to identify neural markers of future affective disorder risk in young adults [15, 19, 27].

Neural networks associated with WM and ER include the central executive network (CEN) [28], which is centered on the dorsolateral prefrontal cortex (dlPFC), lateral posterior parietal cortex (lPPC), and caudate nucleus; the default mode network (DMN) [29] which supports self-referential processing and introspection and is centered on the posterior cingulate cortex (PCC), precuneus, medial prefrontal cortex (mPFC) [29], and inferior parietal cortex; and the salience network (SN) [30] which detects and processes salient, including emotional cues, and is centered on the dorsal anterior cingulate cortex (dACC), anterior insula, and amygdala [28].

We previously demonstrated that during WM, elevated left dlPFC activity, a key node of the CEN, and elevated right precuneus activity, a key node of the DMN, were positively associated with current depression severity in two independent samples of young adults at risk for affective disorders [20]. Elevated left dlPFC activity during a subprocess of ER involving redirection of attention away from emotional distractors during WM was also positively associated with current depression severity. These findings indicate an association between greater current depression severity and greater involvement of the DMN and CEN during WM, and greater involvement of the CEN during an ER subprocess that is dependent on WM [19]. The interpretation of these findings was that in individuals with higher levels of depression severity, greater recruitment of the CEN during WM and ER might be required in order to compensate for greater introspection and self-referential processing, processes subserved by the DMN [29, 31–33]. Furthermore, these associations were specific to depression, and not generalizable to mania/hypomania; and, in a smaller sample, were predictive markers of future depression severity, as assessed 12 months post baseline scan. Additionally, a later study reported positive relationships among the magnitude of right precuneus activity during WM, and magnitude of left dlPFC activity during ER, and a trait measure of future depression risk across three independent young adult samples [21]. Together, these findings provide strong evidence in support of the roles of the right precuneus during WM and left dlPFC during WM and during ER subprocesses dependent on WM as neural markers of present depression severity, and indicate their potential roles as neural markers of future depression risk and severity. Given that longitudinal analytic approaches allow for stronger inferences to be made relative to cross-sectional studies regarding causal relationships [34, 35], our previous longitudinal findings highlighting relationships between neural activity and future depression need to be replicated. This approach will help identify robust and reproducible neural marker predictors of future depression [36].

The goal of the present study was thus to replicate and extend previous findings by examining relationships among DMN (right precuneus) and CEN (left dlPFC) activity during WM and ER tasks and future depression severity in an independent young adult sample. We also aimed to determine the extent to which these findings were evident in a larger combined dataset comprising the original [20] and new samples, in order to assess the robustness of the effect sizes, and generalizability of the neural marker-future depression severity relationships to this larger dataset. Given our previous findings, we hypothesized that: of (1) the magnitudes WM-related left dlPFC and right precuneus activity, and ER-related left dlPFC activity, would be positively associated with future depression severity 12 months post baseline scan in the new sample; (2) findings would be replicated in the larger, combined sample with similar effect sizes; (3) these relationships would be specific to future depressive severity and not associated with future mania/hypomania severity.

## Methods

### Participants

Participants were two independent samples of young adults recruited across a range of subsyndromal to syndromal depression (with and without other, comorbid disorders, e.g., anxiety disorders and ADHD). As this study focused on affective disorder risk/expression i.e. BD and MDD, the sample necessarily included individuals with MDD, given that MDD is in itself a risk factor for BD. The sample also included those with subthreshold symptoms of depression and mania. The original, previously-published sample was harmonized across the original two subsamples (n = 44 with 12 months of follow-up clinical data; n = 33 female, age= 19.28 (1.98)) [20]. The new sample comprised n = 138 young adults, who were scanned on a 3 T Siemens Prisma scanner at baseline (see Methods in the Supplement for parameters); n = 61 were removed due to: missing data (n = 11), excessive motion (>4 mm or mean framewise displacement >0.5) (n = 23), severe artifacts in their neuroimaging data (n = 3), and missing 12 month follow-up data (n = 24). The final sample with 12 month follow-up data was n = 77: 50 female, age = 24.68. The range of HAM-D scores was 0–18 (Table 1). See Supplement Methods for comparison of demographic and clinical between included and excluded participants. The combined sample comprised the original sample and the new sample: n = 121: 83 female, age = 23.81. The range of HAM-D scores was 0–23 (Table 1). Participants in both samples were recruited through student counseling centers, participant registries, and community advertisements. The University of Pittsburgh Institutional Review Board approved this study, and all participants gave written informed consent.

**Table 1:** Demographic and clinical measures of the new and combined samples.

	New Sample n77	Combined Sample n121	Statistics	P-value
Age, mean (SD), years	24.6 (2.89)	23.81 (2.83)	t = 2.08	0.881
Gender, No. (%), female	50 (64.9)	83 (68.6)	X2 = 0.414	0.813
**Clinical measures at 12 month follow-up**				
**HAM-D**				
range	0–18	0–23		
mean (SD)	5.14 (4.60)	6.96 (5.63)	t = −2.36	0.051
**HAM-A**				
range	0–8	0–14		
mean (SD)	2.88 (2.86)	4.57 (4.53)	t = −2.92	<0.001
**YMRS**				
range	0–13	0–21		
mean (SD)	1.16 (1.86)	1.66 (2.38)	t = −1.58	0.061
**Clinical measures at baseline**				
**HAM-D**				
range	0–9	0–30		
mean (SD)	1.38 (0.25)	5.80 (0.66)	t = −5.19	<0.001
**HAM-A**				
range	0–9	0–27		
mean (SD)	1.13 (0.19)	4.66 (0.56)	t = −4.93	<0.001
**YMRS**				
range	0–2	0–9		
mean (SD)	0.26 (0.06)	1.15 (0.16)	t = −4.37	<0.001
**Diagnosis at baseline**				
MDD, No. (%)	0 (0%)	6 (5.0%)	X2 = 3.938	0.047
Anxiety, No. (%)	1 (1.3%)	23 (19.0%)	X2 = 13.855	<0.001
ADHD, No. (%)	2 (2.6%)	6 (5.0%)	X2 = 0.677	0.411
Psychotropic medication use, No. (%)	0 (0%)	2 (1.65%)	X2 = 1.286	0.257
Antidepressants, No.	0	1		
Mood stabilizers, No.	0	1		

No. number, SD standard deviation, MDD Major Depressive Disorder, ADHD Attention-deficit/hyperactivity disorder. Age, gender and clinical measures were assessed at 12 months post-baseline scan. Participants may be diagnosed with more than one disorder.

### Clinical measures

Participants were assessed on several clinical measures (see Supplement). The analyses in the present study focused on depression, assessed using the Hamilton Rating Scale for Depression (HAM-D) [37] and mania/hypomania assessed using the Young Mania Rating Scale (YMRS) [38], each measured at baseline (to assess current symptom severity) and 12 months post scan (to assess future symptom severity). Given that anxiety is highly comorbid with depression, the Hamilton Rating Scale for Anxiety (HAM-A) was used to assess future anxiety severity in exploratory analyses [39].

### Exclusion criteria

Exclusion criteria included: History of head injury, neurological disorders, pervasive developmental disorder, systemic medical disease and treatment; premorbid NAART IQ [40] estimate <85; visual disturbance <20/40 Snellen visual acuity; left/mixed handedness (Annett criteria [41]); history of alcohol/substance use disorder (SUD) and/or illicit substance use (except cannabis at non-SUD levels, given its common usage in young adults [42]) over the last 3 months determined by Structured Clinical Interview for DSM-5 (SCID) [43]; MRI exclusion criteria: metallic objects, claustrophobia, positive pregnancy test for females or self-reported pregnancy; inability to understand English; BD and psychotic disorders.

### Functional imaging task

An adapted emotional n-back working memory task (EFNBACK) designed to measure attentional control over emotional distracters, an emotional regulation subprocess, was used [15, 16, 18–21]. See Methods in the Supplement for task details, neuroimaging data acquisition parameters, and preprocessing steps for neural activity and functional connectivity (FC).

### Regions of interest

We used a single anatomical region of interest (ROI) mask to identify neural regions to test our hypotheses, created from the WFU PickAtlas Wake Forest University, Winston-Salem [44], specifically using the Brodmann Area atlas within the WFU PickAtlas. The mask included gray matter regions in the CEN (bilateral dlPFC (BA9 and BA46), caudate (body, head)), DMN (bilateral precuneus (BA7)) and SN (bilateral insula, dACC (BA24 and BA32), amygdala). The regions chosen are central regions within the CEN, DMN and SN and are most consistently activated by WM and ER tasks [16, 19–21]. The putamen was also included as it is activated in this task due to the motor response required to complete the task [45]. The mask included 14010 voxels (voxel size 2 mm^3^).

### Overarching models

Models were constructed using neural regions corresponding to dlPFC and precuneus regions that previously showed significant relationships with future depression severity in the original sample [20]. See Methods section in the Supplement for details on how extraction was undertaken. To examine future depression severity, the dependent variable (DV) was HAM-D score 12 months post baseline scan. For the WM model, the independent variables (IVs) were WM-related right precuneus activity and left dlPFC activity. For the ER model, the IV was ER-related left dlPFC activity. Baseline (current) depression severity was used as a covariate in all models examining relationships between neural variables and future depression severity. This approach allowed us to assess whether neural measures provide incremental predictive value. To examine future mania/hypomania severity, the dependent variable (DV) was YMRS score 12 months post baseline scan. For the WM model, the independent variables (IVs) were WM-related right precuneus activity and left dlPFC activity. For the ER model, the IV was ER-related left dlPFC activity. Baseline (current) mania/hypomania severity was used as a covariate in all models examining relationships between neural variables and future mania/hypomania severity.

### Data analytic plan

### Aim 1 – Future Depression Severity Prediction in the New Sample

**Step-1**. Following the method previously used for each of the two sub-samples of the original sample [20], models were constructed to examine neural activity and FC during WM: to the 2-back WM condition without face distractors versus the 0-back WM condition without face distractors contrast (2-back WM > 0-back WM); and during redirection of attention away from emotional distracters during WM, i.e., ER: to the 2-back WM condition with emotional face distractors versus the 0-back WM condition without face distractors contrast (2-back ER > 0-back WM). We identified regions showing significant activity and FC in the region of interest (ROI) mask to 2-back WM > 0 back WM and 2-back ER > 0-back WM across participants in SPM12 in the new sample. We extracted parameter estimates of activity and FC for the regions also present in the new sample that had been significantly activated to this contrast in the original sample [20].

**Step-2**. For the DV (future depression severity, HAM-D score 12 months post baseline scan) we assessed the distributional characteristics to identify the appropriate regression model to use in Step 3.

**Step-3**. Using the appropriate regression family in SPSS, we examined in the new sample the extent to which activity in regions corresponding to dlPFC and precuneus regions that previously showed significant relationships with future depression severity in the original sample [20] also showed significant relationships with future depression severity in the new sample. One regression model was used to examine relationships among WM-related neural regional activity and future depression severity, and a separate regression model was used to examine ER-related neural regional activity and future depression severity. Baseline (current) depression severity was used as a covariate in all models. A Benjamini–Hochberg FDR adjusted p value < 0.05 was used to correct for multiple IV-DV comparisons within each IV-DV model.

**Step-4**. For each model, post-hoc analyses were conducted to compute McFadden’s pseudo R-squared, estimating the proportion of variance in the DV explained by the IVs. Two pseudo R-squared values were calculated: one comparing the full model (including all neural and baseline depression severity IVs) with an intercept-only model, and another comparing the full model (all IVs) with a model containing only baseline depression severity.

### Aim 2 – Future Depression Severity Prediction in the Combined Sample

**Step-1**. Using neuroCOMBAT in R to control for scanner effects, we harmonized the extracted parameter estimates of activity and FC in the above regions to WM and ER of the new and original samples into a combined sample.

**Step-2**. For the DV (future depression severity, HAM-D score 12 months post baseline scan) we assessed the distributional characteristics to identify the appropriate regression model to use in Step 3.

**Step-3**. Using the appropriate regression family in SPSS, we examined in the combined sample the extent to which activity in regions corresponding to the dlPFC and precuneus regions that previously showed significant relationships with future depression severity in the original sample [20] also showed significant relationships with future depression severity in the combined sample. One regression model was used to examine relationships among WM-related neural regional activity and future depression severity, and a separate regression model was used to examine ER-related neural regional activity and future depression severity. Baseline (current) depression severity was used as a covariate in all models. A Benjamini–Hochberg FDR adjusted p value < 0.05 was used to correct for multiple IV-DV comparisons within each IV-DV model.

**Step-4**. For each model, post-hoc analyses were conducted to compute McFadden’s pseudo R-squared, estimating the proportion of variance in the DV explained by the IVs. Two pseudo R-squared values were calculated: one comparing the full model (including all neural and baseline depression severity IVs) with an intercept-only model, and another comparing the full model (all neural and baseline depression severity IVs) with a model containing only baseline depression severity.

### Aim 3 – Future Mania/Hypomania Severity Prediction in the New and Combined Samples

**Step-1**. For the DV (future mania/hypomania severity, YMRS score 12 months post baseline scan) we assessed the distributional characteristics to identify the appropriate regression model to use in Step 12.

**Step-2**. Using the appropriate regression family in SPSS, we examined in the new and combined samples whether activity in regions corresponding to the dlPFC and precuneus regions previously identified as significantly related to future depression symptom severity in the original sample [20] also showed significant relationships with future mania/hypomania severity in the new sample. One regression model was used to examine relationships among WM-related neural regional activity and future mania/hypomania severity, and a separate regression model was used to examine ER-related neural regional activity and future mania/hypomania severity. Baseline (current) mania/hypomania severity was used as a covariate in all models. A Benjamini–Hochberg FDR adjusted p value < 0.05 was used to correct for multiple IV-DV comparisons within each IV-DV model.

### Exploratory analyses

Given that anxiety is highly comorbid with depression, exploratory analysis was conducted using the HAM-A as the DV. Post-hoc sensitivity tests of Aim 1 Step-2 were completed after excluding individuals with diagnoses of MDD, anxiety disorders or ADHD at baseline, and medicated individuals. Additional analyses were also conducted in the new sample using a two-step approach involving: 1. inclusion of all significant neural activity and FC measures in the ROI mask as IVs (and future depression / mania/hypomania severity as the DV) in an elastic net penalized regression [46] for IV selection; 2. regression analyses of the selected non-zero IVs from the elasticnet regression (and future depression / mania/hypomania severity as the DV).

Additional analyses were completed without including baseline depression or mania/hypomania as covariates in our models.

Effect size dissimilarity was also examined using Mahalanobis distance.

## Results

### Aim 1 – Future Depression Severity Prediction in the New Sample

**Step-1**. We showed patterns of CEN (dlPFC) and DMN (precuneus) activity during WM (2-backWM >0-backWM), and CEN (dlPFC) activity during ER (2-backER >0-backWM) that were consistent with the original sample (Table 2) [20].

**Table 2:** Corresponding working memory- and emotional regulation-related activity across the new and original samples.

New sample								Original sample, subsample 1								Original sample, subsample 2							
WM								WM								WM							
region	laterality	x	y	z	k	t	p FWE-corr	region	laterality	x	y	z	k	t	p FWE-corr	region	laterality	x	y	z	k	t	p FWE-corr
dlPFC	left	−48	10	30	217	12.19	<0.001	dlPFC	left	−50	10	30	54	9.96	0.003	dlPFC	left	−48	10	30	13	7.16	0.016
dlPFC	left	−44	30	20	77	9.25	<0.001	dlPFC	left	−44	2	30	89	12.60	0.002	dlPFC	left	−44	30	20	10	6.19	0.019
dlPFC	left	−44	28	38	78	7.87	<0.001	dlPFC	left	−46	28	24	16	6.57	<0.001	dlPFC	left	−46	4	30	47	8.12	0.003
precuneus	right	34	−52	50	407	8.92	<0.001	precuneus	right	32	−62	44	332	9.33	<0.001	precuneus	right	34	−52	50	36	7.27	0.005
precuneus	left	−36	−58	54	463	11.44	<0.001	precuneus	left	−26	−70	34	10	9.05	0.021	precuneus	left	−32	−62	48	10	8.04	0.019
dACC	left	−2	8	52	16	9.45	<0.001	dACC	left	−6	6	52	68	8.34	0.002	dACC	left	−2	8	52	90	8.17	0.001
**ER**								**ER**								**ER**							
**region**	**laterality**	**x**	**y**	**z**	**k**	**t**	**p FWE-corr**	**region**	**laterality**	**x**	**y**	**z**	**k**	**t**	**p FWE-corr**	**region**	**laterality**	**x**	**y**	**z**	**k**	**t**	**p FWE-corr**
dlPFC	left	−46	4	34	222	13.63	<0.001	dlPFC	left	−44	2	30	99	12.03	0.002	dlPFC	left	−40	2	28	58	9.73	0.003
dlPFC	left	−44	30	34	107	9.88	<0.001	dlPFC	left	−46	28	24	31	8.62	0.012	dlPFC	left	−44	10	30	27	8.57	0.009
dlPFC	left	−44	30	24	88	10.28	<0.001	dlPFC	left	−48	10	30	54	10.50	<0.001	dlPFC	left	−48	30	20	13	8.78	0.017
dlPFC	right	48	38	30	16	6.18	<0.001	dlPFC	right	44	10	30	88	6.64	0.007	dlPFC	right	46	10	30	31	6.97	0.008
dlPFC	right	46	32	24	30	7.50	0.002	dlPFC	right	42	2	28	16	6.16	0.021	dlPFC	right	48	8	38	59	6.37	0.003
precuneus	right	36	−58	52	417	10.81	<0.001	precuneus	right	12	−70	54	383	10.28	<0.001	precuneus	right	28	−60	44	74	8.81	0.002
precuneus	left	−32	−52	50	468	13.08	<0.001	precuneus	left	−32	−62	44	397	10.89	<0.001	precuneus	left	−32	−62	48	10	8.04	0.019
dACC	left	−4	16	46	42	10.46	<0.001	dACC	left	−6	6	52	79	8.18	0.004	dACC	left	−4	16	46	117	10.46	<0.001

WM contrast is 2-backWM > 0-backWM. ER contrast is 2-backER > 0-backWM. Abbreviations: WM working memory; ER emotional regulation, dlPFC dorsolateral prefrontal cortex, dACC dorsal anterior cingulate cortex, pFWE family-wise error corrected, k cluster size.

**Step-2**. The residuals of the DV, future depression severity (HAM-D score 12 months post baseline scan) showed non-normal distribution with a positive skew, and the standard deviations of the DV was larger than the mean suggesting overdispersion. Given these characteristics we used negative binomial regression models for all analyses [47].

**Step-3**.

**Fig. 1:**
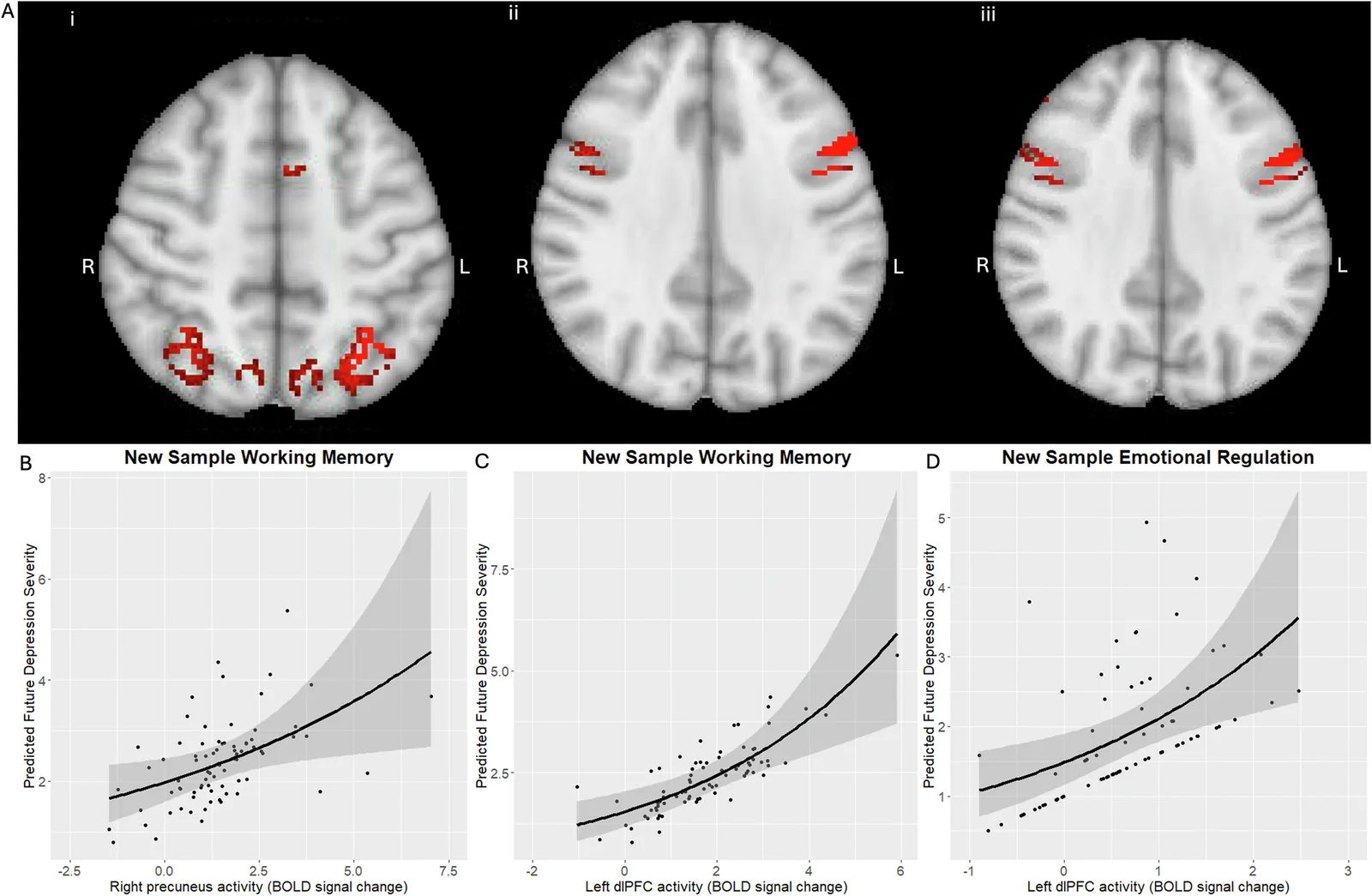
Prediction of future depression severity from candidate neural markers in the new sample. **A** Working memory and emotional regulation related activity during the emotional n-back task in the new sample. i) WM-related precuneus activity. ii) WM-related dorsolateral prefrontal cortex activity. iii) ER-related dorsolateral prefrontal cortex activity. **B** Working memory activity prediction of future depression severity from WM-related right precuneus activity and **C** WM-related left dlPFC activity. **D** Emotional regulation activity prediction of future depression severity from ER-related left dlPFC activity. The scatterplots reflect the relationships between the individual neural variable IV (x axis) and the predicted future depression severity from the full negative binomial model (y axis). In the model in Fig. 1D, the prediction of future depression severity by ER-related neural activity, there was only one neural variable in the model, left dlPFC activity.

**WM:** A negative binomial regression model revealed that right precuneus activity (B = 0.213, CI: 1.055–1.452, p = 0.009; qFDR=0.009) to 2-back WM > 0-back WM was positively associated with future depression severity. A negative binomial regression model revealed that left dlPFC activity (B = 0.478, CI: 1.367–1.903, p < 0.001; qFDR=0.003) to 2-back WM > 0-back WM was positively associated with future depression severity (Table 3A; Fig. 1Ai-ii, B-C).

**Table 3:** **A**. Working memory-related activity predicting Hamilton Depression scale scores 12 months post scan in the new sample. **B**. Emotional regulation-related activity predicting Hamilton Depression scores scale 12 months post scan in the new sample.

Parameter		*B*	Hypothesis test			95% Cl for Exp(*B*)			McFadden’s pseudo R-squared	
			Wald *χ*2	Sig.	FDR	Exp(*B*)	Lower	Upper	Full model	Neural IVs only
**A. Working Memory**										
Baseline depression		0.172	8.153	0.004	0.006	1.187	1.055	1.336	32.17%	
dlPFC	left	0.478	32.038	<0.001	0.003	1.613	1.367	1.903		15.59%
Precuneus	right	0.213	6.862	0.009	0.009	1.238	1.055	1.452		
**B. Emotional Regulation**										
Baseline depression		0.378	44.972	<0.001	0.001	1.459	1.306	1.629	25.43%	
dlPFC	left	0.612	19.283	<0.001	0.001	1.845	1.404	2.424		7.21%

Exp(B) odds ratio or a 1-unit change in predictor variable is an Exp(B) increase in the dependent variable (HAM-D score 12 months post scan), CI confidence interval, dlPFC dorsolateral prefrontal cortex, FDR false discovery rate (Benjamini–Hochberg FDR-corrected adjusted p value < 0.05).

**ER:** A negative binomial regression model revealed that left dlPFC activity (B = 0.612, CI: 1.404–2.424, p < 0.001; qFDR = 0.001) to 2-back ER > 0-back WM was positively associated with future depression severity (Table 3B; Fig. 1Aiii, D). There were no overlapping significant FC patterns between the original and new samples; therefore, replication regression analyses for FC could not be conducted in the new sample.

**Step-4**.

**WM:** A pseudo R-squared, calculated containing all IVs from the model versus an intercept-only model, indicated that 32.17% of the variance in future depression severity was explained by these IVs.

A pseudo R-squared, calculated containing all IVs from the model versus a baseline depression-only model, indicated that 15.59% of the additional variance in future depression severity was explained by these IVs.

**ER:** A pseudo R-squared, calculated containing all IVs from the model versus an intercept-only model, indicated that 25.43% of the variance in future depression severity was explained by these IVs.

A pseudo R-squared, calculated containing all IVs from the model versus a baseline depression-only model, indicated that 7.21% of the additional variance in future depression severity was explained by these IVs.

### Aim 2 – Future Depression Severity Prediction in the Combined Sample

**Step-1**. Consistent patterns of CEN (dlPFC) and DMN (precuneus) activity during WM (2-backWM>0-backWM), and CEN (dlPFC) activity during ER (2-backER >0-backWM) were observed (Table 2), and harmonization of extracted parameter estimates between the original and new samples was successful.

**Step-2**. The residuals of the DV, future depression severity (HAM-D score 12 months post baseline scan) a showed non-normal distribution with a positive skew, and the standard deviations of the DV was larger than the mean suggesting overdispersion. Given these characteristics we used negative binomial regression models for all analyses [47].

**Step-3**.

**Fig. 2:**
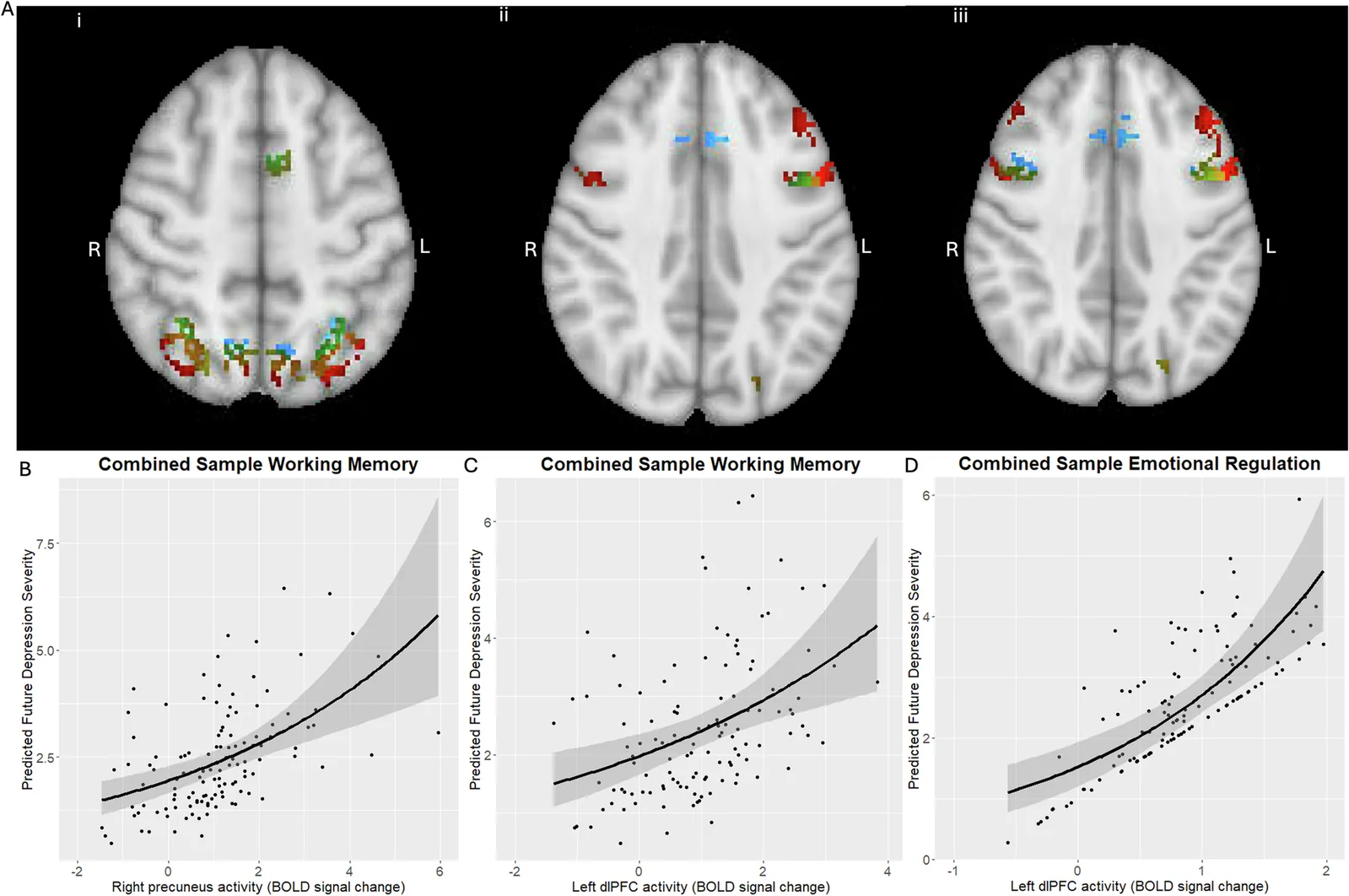
Prediction of future depression severity from candidate neural markers in the combined sample. **A** Working memory and emotional regulation related activity during the emotional n-back task in the new sample (red), original sample subsample 1 (yellow), original sample subsample 2 (blue). Green indicates regions of overlap of all samples. i) WM-related precuneus activity. ii) WM-related dorsolateral prefrontal cortex activity. iii) ER-related dorsolateral prefrontal cortex activity. **B** Working memory activity prediction of future depression severity from WM-related right precuneus activity and **C** WM-related left dlPFC activity. **D** Emotional regulation activity prediction of future depression severity from ER-related left dlPFC activity. The scatterplots reflect the relationships between the individual neural variable IV (x axis) and the predicted future depression severity from the full negative binomial model (y axis). In the model in Fig. 2D, the prediction of future depression severity by ER-related neural activity, there was only one neural variable in the model, left dlPFC activity.

**WM:** A negative binomial regression model revealed that right precuneus activity (B = 0.346, CI: 1.225–1.631, p < 0.001; qFDR=0.001) to 2-back WM > 0-back WM was positively associated with future depression severity. A negative binomial regression model revealed that left dlPFC activity (B = 0.298, CI: 1.152–1.576, p < 0.001; qFDR=0.001) to 2-back WM > 0-back WM was positively associated with future depression severity (Table 4A; Fig. 2Ai-ii, B-C).

**Table 4:** **A**. Working memory-related activity predicting Hamilton Depression scale scores 12 months post scan in the combined sample. **B**. Emotional regulation-related activity predicting Hamilton Depression scale scores 12 months post scan in the combined sample.

Parameter		*B*	Hypothesis test			95% Cl for Exp(*B*)			McFadden’s pseudo R-squared	
			Wald *χ*2	Sig.	FDR	Exp(*B*)	Lower	Upper	Full model	Neural IVs only
**A. Working Memory**										
Baseline depression		0.134	98.195	<0.001	0.001	1.143	1.113	1.174	38.07%	
dlPFC	left	0.298	13.871	<0.001	0.001	1.347	1.152	1.576		7.74%
Precuneus	right	0.346	22.522	<0.001	0.001	1.414	1.225	1.631		
**B. Emotional Regulation**										
Baseline depression		0.088	39.979	<0.001	0.001	1.092	1.063	1.122	42.70%	
dlPFC	left	1.285	102.505	<0.001	0.001	3.616	2.82	4.638		14.63%

Exp(B) odds ratio or a 1-unit change in predictor variable is an Exp(B) increase in the dependent variable (HAM-D score 12 months post scan), CI confidence interval, dlPFC dorsolateral prefrontal cortex, FDR false discovery rate (Benjamini–Hochberg FDR-corrected adjusted p value < 0.05).

**ER:** A negative binomial regression model revealed that left dlPFC activity (B = 1.285, CI: 2.820–4.638, p < 0.001; qFDR = 0.001) to 2-back ER > 0-backWM was positively associated with future depression severity (Table 4B; Fig. 2Aiii, D).There were no overlapping significant FC patterns between the original and combined samples, therefore, replication regression analyses for FC could not be conducted in the combined sample.

**Step-4**.

**WM:** A pseudo R-squared, calculated containing all IVs from the model versus an intercept-only model, indicated that 38.07% of the variance in future depression severity was explained by these IVs.

A pseudo R-squared, calculated containing all IVs from the model versus a baseline depression-only model, indicated that 7.74% of the additional variance in future depression severity was explained by these IVs.

**ER:** A pseudo R-squared, calculated containing all IVs from the model versus an intercept-only model, indicated that 42.70% of the variance in future depression severity was explained by these IVs.

A pseudo R-squared, calculated containing all IVs from the model versus a baseline depression-only model, indicated that 14.63% of the additional variance in future depression severity was explained by these IVs.

### Aim 3 – Future Mania/Hypomania Severity Prediction in the New and Combined Samples

**Step-1**. The residuals of the DV, future depression severity (YMRS score 12 months post baseline scan) a showed non-normal distribution with a positive skew, and the standard deviations of the DV was larger than the mean suggesting overdispersion. Given these characteristics we used negative binomial regression models for all analyses [47].

**Step-2**. Neither right precuneus nor left dlPFC activity during WM or left dlPFC activity during ER were significantly associated with future mania/hypomania severity in the new sample (Supplemental Table S1). Neither right precuneus nor left dlPFC activity during WM or left dlPFC activity during ER were significantly associated with future mania/hypomania severity in the combined sample (Supplemental Table S2).

### Exploratory analyses

See Supplement for, future anxiety severity results (Supplemental Tables S3–4), the additional analyses using a two-step approach (Supplemental Table S5–6), and sensitivity analyses excluding individuals with diagnoses of MDD, anxiety disorders or ADHD at baseline, and medicated individuals (Supplemental Tables S7–S12).

When using the additional two-step approach, left rather than right precuneus activity was significantly related to future depression severity in the new sample (B = 0.024) (Supplemental Table S5).

Findings remained unchanged when analyses were repeated without including baseline depression or mania/hypomania as covariates in the models (Supplementary Tables S13–S16). See supplement for effect size dissimilarity results.

## Discussion

Building on prior work demonstrating robust positive associations between left dlPFC activity and right precuneus activity during WM, as well as left dlPFC activity during ER, and depression severity up to 12 months post baseline scan [20], the present study aimed to replicate these findings in a new, independent sample of young adults recruited across a range of subsyndromal to syndromal depression, and thus across a range of risk for new affective disorders. We replicated our previous findings, showing that left dlPFC and right precuneus activity during WM, as well as left dlPFC activity during ER, were associated with depression severity 12 months post baseline scan in the new sample as well as in a combined sample comprising the new and original samples [20]. Importantly, these effects were specific to depression severity, and were not observed in relation to future mania/hypomania severity, at 12 months after baseline scan. These findings also align with another previous study in which right precuneus activity during WM and left dlPFC activity during ER were associated with lifetime depression risk, as measured by the Mood Spectrum Self-Report Lifetime Questionnaire (MOODS-SR-L) [21]. Together, these replicated, longitudinal findings strengthen evidence that higher levels of activity in DMN and CEN regions during WM and ER are candidate neural markers of future depression severity.

Elevated right precuneus activity, a key DMN region in which activity is often suppressed during goal-directed tasks, has been associated with difficulty disengaging from internally-focused, ruminative thought [29, 31–33] in depressed individuals [48–50]. While rumination was not directly measured in the current study, the observed association between WM-related DMN activity and future depression severity is consistent with prior work linking altered DMN function to self-referential processing and depressive symptoms [51–56]. This interpretation aligns with an extensive literature reporting associations between higher levels of rumination with greater depressive symptom severity [57], increased risk of episode onset [51, 58], longer illness duration [48, 58], and the emergence of depressive symptoms in initially non-depressed individuals [54]. Together with the observed association between elevated left dlPFC activity during WM and future depression severity, one possible interpretation is that higher levels of intrusive, self-referential or ruminative processes might result in compensatory, higher levels of CEN dlPFC activity [59] during goal-directed tasks such as WM in individuals with greater future depression severity.

Alternative explanations can be considered regarding dlPFC and precuneus activity as predictors of future depression severity. Elevated/aberrant dlPFC activity may reflect inefficiencies in CEN functioning that predispose to depressive traits and symptoms and contribute to reduced DMN suppression. Additionally, consistent with longitudinal and cross-network findings from Rumination-Focused Cognitive Behavioral Therapy trials targeting rumination in adolescents and youth with a history of depression [60, 61], disrupted functional connectivity between dlPFC and posterior DMN regions (e.g., PCC/precuneus), rather than regional hyperactivity alone, might confer risk for worsening depressive symptoms. Additionally, while we did not formally test hemispheric laterality of precuneus activity in the new sample as the goal of this study was to directly test the previously identified right precuneus finding from the original study, when using the additional two-step approach in the new sample, bilateral precuneus activity was related to future depression severity, but only the cluster in the left precuneus reached significance (Supplemental Table S5). This concurs with previous findings showing that both right and left precuneus activity are associated with ruminative, self-referential processing [60, 61].

Similarly, during ER, elevated left dlPFC activity was positively associated with greater depression severity 12 months post baseline scan, replicating prior observations of abnormally elevated dlPFC activity in depressed individuals during ER tasks [16, 17, 21, 62]. This finding suggests that higher levels of CEN recruitment might be necessary to maintain ER performance in individuals with greater future depression severity. By replicating these associations longitudinally in an independent sample, our findings provide strong evidence that elevated DMN and CEN activity [28] during WM and ER are candidate neural markers of cognitive processes that reflect vulnerability to future depression, and addresses concerns about neuroimaging study variability [36]. Furthermore, identifying robust and reproducible candidate neural marker predictors of future depression not only provides neural markers to aid early identification of at-risk individuals, but also provides neural targets to guide the development of new interventions to delay or even halt the onset and/or worsening of future depression in these individuals.

The effect sizes (pseudo R-squared values) for the full models including all IVs in both the new and the combined samples ranged from approximately 0.25–0.43. These effect sizes are relatively large [63] and parallel findings in other studies examining neural markers of future affective psychopathology [64, 65]. Even for neural IVs alone, in the new sample, left dlPFC and right precuneus activity during WM explained 15.59% of the variance in future depression severity; and in the combined sample, left dlPFC activity alone during ER explained 14.63% of the variance in future depression severity. Additional analyses of effect size dissimilarity, as measured by Mahalanobis distance, are provided in the Supplement. Together, these findings highlight the role of left dlPFC activity in particular during WM and ER as a predictive marker of future depression severity.

The left dlPFC is a key target for neuromodulation in individuals with treatment-resistant depression [66]. Recent research using the Stanford Accelerated Intelligent Neuromodulation Therapy (SAINT) protocol [67–69], spanning open-label studies and subsequent double-blind controlled trials, demonstrates rapid symptom improvement and high remission rates using high-dose, connectivity-guided intermittent theta-burst stimulation (iTBS) delivered to functionally defined left dlPFC targets. This reflects a precision-medicine approach that contrasts with the traditional assumption of a uniform dlPFC coordinate. Inter-individual variability in treatment response, particularly evidence that stronger baseline dlPFC–subgenual anterior cingulate cortex (sgACC) anticorrelation predicts greater therapeutic benefit, suggests, however, that some individuals may benefit from neuromodulation interventions at alternative targets, or parameter adjustments. Furthermore, these findings suggest that in individuals with persistent depression but with heightened levels of left dlPFC activity during WM and/or ER, the CEN might be functioning near or at full capacity. For these individuals, efforts to further increase left dlPFC activity with neuromodulation might therefore be of little benefit, and could have a detrimental effect of overburdening CEN capacity rather than having a therapeutic effect of improving cognitive functions supporting WM and ER via CEN upregulation. This line of reasoning further emphasizes the importance of considering inter-individual variation in activity in regions such as the left dlPFC prior to neuromodulation, and other, interventions, in order to tailor such interventions appropriately to either reduce maladaptive, or upregulate dysregulated, patterns of activity in neural networks implicated in cognitive processes important for ER.

This study had limitations. None of the FC patterns in the original sample [20] was present in the new sample and thus relationships between FC in neural regions of interest and future depression severity could not be tested for replication. While some participants were medicated or had depressive disorders, anxiety disorders, or ADHD at baseline, findings remained the same after exclusion of individuals with depressive and anxiety disorders in both the new and combined samples, and medicated individuals in the combined sample (Supplemental Tables S7–S12). Exclusion of individuals with ADHD resulted in the association between right precuneus activity and future depression severity becoming near-significant, rather than significant in the new sample (Supplemental Table S8). Given the high correlation between HAM-D and HAM-A scores (Supplement), we conducted additional regression analyses using HAM-A scores 12 months post baseline scan as the DV and observed findings consistent with those obtained for HAM-D scores (Supplemental Tables S3–4). Finally, while the proportion of variance explained by neural predictors is comparable to prior neuroimaging studies of affective risk, these findings should be cautiously interpreted as candidate neural correlates of risk that require further study. Future studies with larger samples, complementary and additional mechanisms of risk, and more time points will be able to examine symptom trajectories more comprehensively. The study, however, had several strengths, including its longitudinal design, the replication of findings, and large sample sizes. Although recruitment strategies differed slightly in the new and original samples (see Methods for new sample and Bertocci et al. [20] for original sample), we observed consistent patterns of findings across samples. In addition, the similarity in the range of future depression severity DVs between the new and combined samples provided an opportunity to evaluate generalizability of findings. Furthermore, when we used an alternative (de novo) two-step approach involving elasticnet for variable selection [46] followed by regression analyses of the selected non-zero variables in the new sample, we observed a very similar pattern of findings for future depression severity, though less so for future anxiety severity (Supplemental Tables S3–4).

We demonstrate a replication of longitudinal relationships between left dlPFC activity and right precuneus activity during WM, and left dlPFC activity during ER, and future depression severity one year later, in a new independent sample, and in a large, combined sample, comprising the new sample and a previously-published sample, of young adults recruited across a range of risk for future affective disorders. These findings provide candidate neural markers to guide risk identification and targeting of new, e.g., neuromodulation, interventions, and highlight the importance of considering baseline levels of neural activity during decision-making about appropriate neuromodulation approaches for individuals with and those at risk for future affective disorders.

## Supplementary information

Supplement

## Data Availability

The dataset is available per request. Please request access by contacting the corresponding author Yvette Afriyie-Agyemang.
