# Supplementary material for: Neural markers of working memory and emotional regulation are predictors of future depression severity in at risk young adults: a replication study
Source: Mol Psychiatry. 2026 Jul 31;31(12):7186–95. doi: 10.1038/s41380-026-03771-5 (PMC13736626; doi:10.1038/s41380-026-03771-5)
Supplement: Supplementary file 1 [file 41380_2026_3771_MOESM1_ESM.docx]

**Methods.** Clinical Measures, Clinical differences between included and excluded participants, Power calculation, Functional Imaging Task, Neuroimaging Data Acquisition, Neuroimaging Data Preprocessing, First-level Modeling, Extraction of Neural Measures, Additional Analyses, Effect size dissimilarity.

Clinical Measures

Scales included the Hamilton Rating Scale for Depression^1^, Hamilton Anxiety Rating Scale^2^, the Young Mania Rating Scale^3^ Snaith-Hamilton Pleasure Scale (SHAPS)^4^, The Brief Psychiatric Rating Scale Expanded Version (BPRS-E; original^5^expanded^6^, Kessler Psychological Distress Scale (K10)^7^, Positive And Negative Affect Scale (PANAS-I)^8^, Trauma History Questionnaire (THQ)^9^, Mood and Anxiety Symptom Questionnaire (MASQ)^10^, Monetary Choice Questionnaire (MCQ), Quality of Life Enjoyment and Satisfaction Questionnaire-Short Form (Q-LES-Q-SF)^11^, Spielberger State & Trait Anxiety Inventory (STAIY-1)^12^. The Neuroticism Extroversion Openness Five Factor Inventory (NEO PI-R)^13^, Structured Clinical Interview for DSM-5 (SCID)^14^, Barratt Simplified Measure of Social Status (BSMSS)^15^, North American National Adult Reading Test NAART^16^, The Mini-Mental Status Examination, MMSE^17^, Behavioral Handedness Index (BHI)^18^, the Longitudinal Interval Follow-up Evaluation (LIFE) Adaptation for Longitudinal Outcome^19^, The Longitudinal Interval Follow-Up Evaluation-Range of Impaired Functioning (LIFE-RIFT)^24^, Barratt Impulsiveness Scale (BIS-11)^20^, Behavioral Inhibition and Activation Scales (BIS/BAS)^21^ , Impulsive Behavior Scale (UPPS-P)^22^, Cigarette/Nicotine Dependence Scale (CDS).

Clinical differences between included and excluded participants

The included and excluded participants in the new sample did not differ on age (t=-.752, p=.631), gender (x2=.939, p=.625), baseline MDD diagnosis (x2=.775, p=.379) or baseline anxiety disorder diagnosis (x2=.766, p=.382).

They differed slightly on baseline ADHD diagnosis (x2=3.813, p=.051) as well as baseline HAM-D (t=1.320, p=.013), HAM-A (t=1.451, p=.012) and YMRS scores (t=1.769, p<.001).

Power calculation

With our new sample size of n=77 for continuous outcome measures, we have 80% power, to detect an FDR corrected to a p-value of 0.05 medium effect for the independent variables.

With our combined sample size of n=121 for continuous outcome measures, we have 80% power, to detect an FDR corrected to a p-value of 0.05 medium effect for the independent variables.

Functional imaging task

An adapted emotional n-back working memory task (EFNBACK) designed to measure attentional control over emotional distracters, an emotional regulation subprocess, was used^23-28^. There are two memory load conditions: a 0-back, no-memory load (e.g., press the button to “M”) and 2-back, high memory load (e.g. press the button whenever the letter is identical to the letter presented in two trials back (L-X-L)), flanked by two face distracters. Each trial comprises a letter flanked with either no pictures, or identical pictures of an actor’s facial expression (fearful, happy, neutral). Therefore, the task has a two factor design: high-memory load (2-back) with and without face distractors, and low-memory load (0-back) with and without face distractors. The task comprises two, approximately 7- min runs. Each run is 8 blocks and each block includes 12 trials. Trial duration is 500 ms. The inter-trial interval comprises a fixation cross (flanked with faces) and is jittered (mean duration= 3500 ms). Participants respond as quickly as possible with their index finger to the target letter. Brief instructions are presented on the screen for 4000 ms at the beginning of each block. Detailed instructions are provided during task practice prior to the scanning session. Incorrect trials were excluded from the analysis.

Neuroimaging Data Acquisition

In the new sample and original sample subsample 2, data were collected using a 3.0 Tesla Siemens Prisma MRI scanner. For the new, mean BOLD T2*-weighted images were collected with a multiband multi-echo EPI (MBME)5-factor sequence covering 12x(MBME)5(=70) oblique slices (TR=1500 ms, TE1=15.60 ms, TE2=39.78 ms, TE3=63.96 ms; 2 mm^3^ slices; FOV=210x210; matrix=96x96; slice thickness=2 mm; flip angle=70°, bandwidth=2290 Hz/Px), along with structural 3D sagittal MPRAGE images (TR/TE=1520/3.17 ms; FOV=256×256mm; flip angle=8°; 1 mm^3^ isotropic voxels; 176 continuous slices) and fieldmaps (TR=731 ms, TE1=4.92 ms, TE2=7.38 ms; flip angle=50°; bandwidth=566 Hz/Px). For original sample subsample 2, BOLD images were acquired with a gradient echo EPI multiband (MB)3 sequence covering 18x(MB)3(=54) oblique slices (TR=1500 ms, TE=30 ms; 2.3 mm^3^ slices; flip angle 55°; FOV=220x220; matrix=96x96). Structural 3D axial MPRAGE images were acquired with the following parameters (TR=1520 ms, TE=3.17 ms; flip angle 8°; FOV=256x256 mm; 1 mm^3^ isotropic voxels; 176 continuous slices).

In original sample subsample 1, data were collected using a 3.0 Tesla Siemens Trio 2 MRI scanner in the Magnetic Resonance Research Center (MRRC) at the University of Pittsburgh Medical Center. A total of 504 blood-oxygenation-level-dependent (BOLD) images were acquired with a simultaneous multi-slice (SMS) gradient echo EPI sequence (18 slices, SMS factor=3, TR=1500, TE=30 ms, Field of View (FOV)=220 × 220 mm, matrix=96 × 96, Flip Angle=55°, Bandwidth= 1860 Hz/Px). In addition, we acquired structural 3D axial MPRAGE images (TR=1500 ms, TE=3.19 ms, Flip Angle 8°, FOV=256 × 256 mm, 1 mm isotropic voxels, 176 continuous slices), and fieldmaps (TR=500 ms, TE1=4.92 ms, TE2=7.38 ms, FOV=220 × 220 mm, matrix=96 × 96, Flip Angle=45°, Bandwidth=1302 Hz/Px).

Neuroimaging Data Preprocessing

Imaging data for the new sample was preprocessed using fMRIPrep^29^, FSL (5.0.9), and AFNI^30^ 20160207 (RRID:SCR_005927). The first volume of the time series and its skull-stripped version were generated from the shortest echo of the BOLD run using fMRIPrep, followed by co-registration to the T1-weighted reference image using FreeSurfer bbregister and estimation of head-motion parameters using FSL mcflirt. Slice-time correction was performed using AFNI 3dTshift. BOLD time-series were then resampled into native space. Multi-echo (ME) independent components analysis (ME-ICA)^31, 32^ was then applied to preprocessed BOLD images using Tedana ^33^for ME integration. Despiking using AFNI 3dDespike and spatial smoothing using a FWHM of 6 mm were then applied.

Data for both original sample subsamples 1 and 2 were preprocessed using a combination of software packages (SPM, FSL^34, 35^, and AFNI) implemented in Nipype^36^. Standard preprocessing steps were applied including realignment to the first volume of the time series, co-registration of realigned images to each participant’s anatomic scan, normalization of anatomic images to the MNI152 template, despiking using AFNI 3dDespike, and spatial smoothing using a Full-width at half maximum (FWHM) of 6 mm, using SUSAN in FSL.

First-level Modeling

SPM12 (Discovery sample) and SPM8 (Test sample 1 and Test sample 2) and were used to generate a first-level fixed-effects general linear model for each participant. The model included six motion parameters and a regressor controlling for physiologic noise derived from the mean signal of white matter and CSF as covariates of no interest. A high-pass frequency filter (256 seconds) and autoregressive modeling (FAST for Discovery sample, AR(1) for Test sample 1 and Test sample 2) were also applied.

Extraction of Neural Measures

A first-level single subject statistical parametric map was generated for the 2-back working memory *without* distractors versus 0-back *without* distractors contrast and 2-back working memory *with* emotional faces distractors (2-backEmWM) versus the 0-back *without* distractors contrast and used in a second-level (group) analysis to identify neural activity across participants during face emotion processing. We extracted the first eigenvariate from individual contrast images for all significant clusters, using a family-wise error (FWE) correction threshold of α= .05, in an emotion regulation mask.

Additional analyses

**Step-1.** Following extraction of parameter estimates (Aim 1, Step-1) in the new sample, elastic net variable selection models were tested with a negative binomial family using the GLMNET package in R^37^ . WM contrast-related and ER contrast-related neural measures from Aim 1 Step-1, age, gender and baseline scores were independent variables (IVs), and HAM-D, HAM-A and or YMRS scores were dependent variables (DVs). There were a total of six IV-DV models: WM-future depression severity, ER-future depression severity, WM-future mania/hypomania severity, ER-future mania/hypomania severity, WM-future anxiety severity and ER-future anxiety severity.

**Step-2**. Using SPSS, negative binomial regression identified significant relationships among WM contrast-related neural, ER contrast-related neural, and demographic measures that were selected as non-zero coefficients associated with the DV in each model in the new sample. A Benjamini-Hochberg FDR adjusted p-value <.05 was used to correct for multiple IV-DV comparisons within each of the IV-DV models.

Effect size dissimilarity

To quantify model-level effect-size dissimilarity between the new and combined samples, Mahalanobis distances were computed using vectors of negative binomial regression coefficients (B values) across predictors. Larger distances indicate greater dissimilarity in multivariate effect profiles. The Mahalanobis distance provides a multivariate measure of dissimilarity between two sets of regression coefficients while accounting for the precision and covariance structure of the parameter estimates. In this study, it quantifies the extent to which the pattern of regression effects observed in the new sample differs from that observed in the combined sample.

**1. Working Memory (WM)**

**Difference vector of the regression estimates of the two samples:**

$$d=\left[ \begin{aligned} 0.038 \\ 0.180 \\ -0.133 \end{aligned} \right]$$

**New Sample Covariance Matrix (**$\boldsymbol{C}$**)**

$$C=\left[ \begin{matrix} 0.00362 & -0.00140 & -0.00143 \\ -0.00140 & 0.00713 & -0.00301 \\ -0.00143 & -0.00301 & 0.00663 \end{matrix} \right]$$

**Combined Sample Covariance Matrix (**$\boldsymbol{D}$**)**

$$D=\left[ \begin{matrix} 0.00018 & -0.00025 & -0.00018 \\ -0.00025 & 0.00640 & -0.00237 \\ -0.00018 & -0.00237 & 0.00532 \end{matrix} \right]$$

**Average covariance matrix of the two samples:**

$$A=\frac{C+D}{2}=\left[ \begin{matrix} 0.00190 & -0.000825 & -0.000805 \\ -0.000825 & 0.006765 & -0.002690 \\ -0.000805 & -0.002690 & 0.005975 \end{matrix} \right]$$

**Mahalanobis distance:**

$$\parallel d\parallel_{A}=\sqrt{d^{T}A^{-1}d}$$

**Result:**

$$\boxed{\parallel d\parallel_{A,WM}\approx2.63}$$

**2. Emotional Regulation (ER)**

**Difference vector of the regression estimates of the two samples:**

$$d=\left[ \begin{aligned} 0.290 \\ -0.673 \end{aligned} \right]$$

**New Sample Covariance Matrix (**$\boldsymbol{C}$**)**

$$C=\left[ \begin{matrix} 0.00317 & -0.00237 \\ -0.00237 & 0.01944 \end{matrix} \right]$$

**Combined Sample Covariance Matrix (**$\boldsymbol{D}$**)**

$$D=\left[ \begin{matrix} 0.00019 & -0.00097 \\ -0.00097 & 0.01612 \end{matrix} \right]$$

**Average covariance matrix:**

$$A=\frac{C+D}{2}=\left[ \begin{matrix} 0.00168 & -0.00167 \\ -0.00167 & 0.01778 \end{matrix} \right]$$

**Mahalanobis distance:**

$$\parallel d\parallel_{A}=\sqrt{d^{T}A^{-1}d}$$

**Result:**

$$\boxed{\parallel d\parallel_{A,ER}\approx7.70}$$

**Results.** Effect size dissimilarity, Additional Analyses.

Effect size dissimilarity

| **Model** | **Mahalanobis Distance** |
| --- | --- |
| Working Memory (WM) | **2.63** |
| Emotional Regulation (ER) | **7.70** |

The ER models exhibited greater multivariate dissimilarity between the new and combined samples than the WM models, as measured by the Mahalanobis distance. This indicates that the multivariate configuration of regression effects was less consistent across samples for the ER model relative to the WM model.

Additional analyses

**Table S1. A.** Working memory-related activity predicting Young Mania Rating scale scores 12 months post scan in the new sample. **B.** Emotional regulation-related activity predicting Young Mania Rating scale scores 12 months post scan in the new sample.

| **Parameter** | | ***B*** | **Hypothesis test** | | **95% Cl for Exp(*B*)** | |  |
| --- | --- | --- | --- | --- | --- | --- | --- |
|  |  |  | **Wald *χ*2** | **Sig.** | **Exp(*B*)** | **Lower** | **Upper** |
| **A. Working Memory** | |  |  |  |  |  |  |
| Baseline mania/hypomania | | 0.293 | 1.376 | 0.241 | 1.341 | 0.821 | 2.189 |
| dlPFC | left | -0.049 | 0.194 | 0.659 | 0.952 | 0.766 | 1.184 |
| Precuneus | right | 0.115 | 0.974 | 0.324 | 1.122 | 0.893 | 1.409 |
| **B. Emotional Regulation** | | |  |  |  |  |  |
| Baseline mania/hypomania | | 0.307 | 1.682 | 0.195 | 1.359 | 0.855 | 2.162 |
| dlPFC | left | 0.154 | 0.777 | 0.378 | 1.167 | 0.828 | 1.644 |

Exp(B) odds ratio or a 1-unit change in predictor variable is an Exp(B) increase in the dependent variable (HAM-D score 12 months post scan), CI confidence interval, dlPFC dorsolateral prefrontal cortex.

**Table S2. A.** Working memory-related activity predicting Young Mania Rating scale scores 12 months post scan in the combined sample. **B.** Emotional regulation-related activity predicting Young Mania Rating scale scores 12 months post scan in the combined sample.

| **Parameter** | | ***B*** | **Hypothesis test** | | **95% Cl for Exp(*B*)** | |  |
| --- | --- | --- | --- | --- | --- | --- | --- |
|  |  |  | **Wald *χ*2** | **Sig.** | **Exp(*B*)** | **Lower** | **Upper** |
| **A. Working Memory** | |  |  |  |  |  |  |
| Baseline mania/hypomania | | 0.214 | 12.304 | 0.000 | 1.238 | 1.099 | 1.396 |
| dlPFC | left | 0.073 | 0.426 | 0.514 | 1.076 | 0.864 | 1.340 |
| Precuneus | right | 0.103 | 1.080 | 0.299 | 1.108 | 0.913 | 1.346 |
| **B. Emotional Regulation** | | |  |  |  |  |  |
| Baseline mania/hypomania | | 0.224 | 14.175 | 0.000 | 1.251 | 1.113 | 1.406 |
| dlPFC | left | 0.207 | 2.535 | 0.111 | 1.230 | 0.953 | 1.586 |

Exp(B) odds ratio or a 1-unit change in predictor variable is an Exp(B) increase in the dependent variable (HAM-D score 12 months post scan), CI confidence interval, dlPFC dorsolateral prefrontal cortex.

**Table S3. A.** Working memory-related activity predicting Hamilton Anxiety scale scores 12 months post scan in the new sample. **B.** Emotional regulation-related activity predicting Hamilton Anxiety scale scores 12 months post scan in the new sample.

| **Parameter** |  | ***B*** | **Hypothesis test** | | **95% Cl for Exp(*B*)** | |  |
| --- | --- | --- | --- | --- | --- | --- | --- |
|  |  |  | **Wald *χ*2** | **Sig.** | **Exp(*B*)** | **Lower** | **Upper** |
| **A. Working Memory** |  |  |  |  |  |  |  |
| Baseline anxiety |  | 0.220 | 7.668 | 0.006 | 1.246 | 1.066 | 1.456 |
| dlPFC | left | 0.205 | 4.702 | 0.030 | 1.227 | 1.02 | 1.477 |
| Precuneus | right | 0.183 | 3.455 | 0.063 | 1.201 | 0.99 | 1.457 |
| **B. Emotional Regulation** |  |  |  |  |  |  |  |
| Baseline anxiety |  | 0.394 | 26.203 | <.001 | 1.484 | 1.276 | 1.726 |
| dlPFC | left | 0.273 | 2.900 | 0.089 | 1.314 | 0.960 | 1.800 |

Exp(B) odds ratio or a 1-unit change in predictor variable is an Exp(B) increase in the dependent variable (HAM-D score 12 months post scan), CI confidence interval, dlPFC dorsolateral prefrontal cortex.

**Table S4. A.** Working memory-related activity predicting Hamilton Anxiety scale scores 12 months post scan in the combined sample. **B.** Emotional regulation-related activity predicting Hamilton Anxiety scale scores 12 months post scan in the combined sample.

| **Parameter** |  | ***B*** | **Hypothesis test** | | **95% Cl for Exp(*B*)** | |  |
| --- | --- | --- | --- | --- | --- | --- | --- |
|  |  |  | **Wald *χ*2** | **Sig.** | **Exp(*B*)** | **Lower** | **Upper** |
| **A. Working Memory** |  |  |  |  |  |  |  |
| Baseline anxiety |  | 0.135 | 60.05 | <.001 | 1.144 | 1.106 | 1.184 |
| dlPFC | left | 0.188 | 4.714 | 0.030 | 1.207 | 1.018 | 1.431 |
| Precuneus | right | 0.275 | 11.585 | <.001 | 1.316 | 1.124 | 1.542 |
| **B. Emotional Regulation** |  |  |  |  |  |  |  |
| Baseline anxiety |  | 0.097 | 31.481 | <.001 | 1.102 | 1.065 | 1.14 |
| dlPFC | left | 0.905 | 47.978 | <.001 | 2.472 | 1.913 | 3.193 |

Exp(B) odds ratio or a 1-unit change in predictor variable is an Exp(B) increase in the dependent variable (HAM-D score 12 months post scan), CI confidence interval, dlPFC dorsolateral prefrontal cortex.

**Table S5. A.** Working memory-related activity predicting Hamilton Depression scale scores 12 months post scan in the new sample, following elastic net for variable selection. **B.** Emotional regulation-related activity predicting Hamilton Depression scale scores 12 months post scan in the new sample, following elastic net for variable selection.

| **Parameter** |  | ***B*** | **Hypothesis test** | | **95% Cl for Exp(*B*)** | |  |
| --- | --- | --- | --- | --- | --- | --- | --- |
|  |  |  | **Wald *χ*2** | **Sig.** | **Exp(*B*)** | **Lower** | **Upper** |
| **A. Working Memory** |  |  |  |  |  |  |  |
| Baseline depression |  | 0.169 | 7.618 | 0.006 | 1.185 | 1.050 | 1.336 |
| dlPFC | left | 0.339 | 11.244 | <.001 | 1.403 | 1.151 | 1.710 |
| Precuneus | right | 0.085 | 0.783 | 0.376 | 1.089 | 0.902 | 1.314 |
| Precuneus | left | 0.278 | 5.123 | 0.024 | 1.321 | 1.038 | 1.681 |
| **B. Emotional Regulation** |  |  |  |  |  |  |  |
| Baseline depression |  | 0.118 | 3.351 | 0.067 | 1.125 | 0.992 | 1.276 |
| dlPFC | left | 0.452 | 5.034 | 0.025 | 1.571 | 1.059 | 2.330 |
| Precuneus | right | 0.112 | 0.251 | 0.616 | 1.119 | 0.721 | 1.735 |
| Precuneus | left | 0.329 | 1.924 | 0.165 | 1.389 | 0.873 | 2.210 |
| dACC- left dACC |  | 0.468 | 6.739 | 0.009 | 1.597 | 1.122 | 2.274 |

Exp(B) odds ratio or a 1-unit change in predictor variable is an Exp(B) increase in the dependent variable (HAM-D score 12 months post scan), CI confidence interval, dlPFC dorsolateral prefrontal cortex.

**Table S6. A.** Working memory-related activity predicting Hamilton Anxiety scale scores 12 months post scan in the new sample, following elastic net for variable selection. **B.** Emotional regulation-related activity predicting Hamilton Anxiety scale scores 12 months post scan in the new sample, following elastic net for variable selection.

| **Parameter** |  | ***B*** | **Hypothesis test** | | **95% Cl for Exp(*B*)** | |  |
| --- | --- | --- | --- | --- | --- | --- | --- |
|  |  |  | **Wald *χ*2** | **Sig.** | **Exp(*B*)** | **Lower** | **Upper** |
| **A. Working Memory** |  |  |  |  |  |  |  |
| Baseline anxiety |  | 0.215 | 7.313 | 0.007 | 1.240 | 1.061 | 1.450 |
| dlPFC | left | 0.092 | 0.633 | 0.426 | 1.096 | 0.875 | 1.373 |
| Precuneus | right | 0.081 | 0.503 | 0.478 | 1.084 | 0.867 | 1.356 |
| Precuneus | left | 0.222 | 2.615 | 0.106 | 1.249 | 0.954 | 1.636 |
| **B. Emotional Regulation** |  |  |  |  |  |  |  |
| Baseline anxiety |  | 0.211 | 6.213 | 0.013 | 1.235 | 1.046 | 1.458 |
| dlPFC | left | 0.256 | 1.278 | 0.258 | 1.291 | 0.829 | 2.011 |
| Precuneus | left | 0.375 | 3.139 | 0.076 | 1.455 | 0.961 | 2.203 |
| dACC- left dACC |  | 0.043 | 0.047 | 0.828 | 1.044 | 0.709 | 1.537 |

Exp(B) odds ratio or a 1-unit change in predictor variable is an Exp(B) increase in the dependent variable (HAM-D score 12 months post scan), CI confidence interval, dlPFC dorsolateral prefrontal cortex.

- No variables were selected as non-zero by the elastic net model for the Young Mania Rating Scale, so subsequent regression analyses could not be completed.
- No individuals had a diagnosis of MDD in the new sample, so subsequent sensitivity analyses could not be completed.

**Table S7. A.** Working memory-related activity predicting Hamilton Depression scale scores 12 months post scan in the new sample after excluding individuals with an anxiety disorder diagnosis at baseline. **B.** Emotional regulation-related activity predicting Hamilton Depression scale scores 12 months post scan in the new sample after excluding individuals with an anxiety diagnosis at baseline.

| **Parameter** | | ***B*** | **Hypothesis test** | | **95% Cl for Exp(*B*)** | | |
| --- | --- | --- | --- | --- | --- | --- | --- |
|  |  |  | **Wald *χ*2** | **Sig.** | **Exp(*B*)** | **Lower** | **Upper** |
| **A. Working Memory** | |  |  |  |  |  |  |
| Baseline depression | | 0.167 | 7.545 | 0.006 | 1.181 | 1.049 | 1.331 |
| dlPFC | left | 0.478 | 32.075 | <.001 | 1.612 | 1.367 | 1.902 |
| Precuneus | right | 0.213 | 6.912 | 0.009 | 1.238 | 1.056 | 1.451 |
| **B. Emotional Regulation** | | |  |  |  |  |  |
| Baseline depression | | 0.372 | 42.116 | <.001 | 1.450 | 1.296 | 1.623 |
| dlPFC | left | 0.613 | 19.405 | <.001 | 1.846 | 1.406 | 2.426 |

Exp(B) odds ratio or a 1-unit change in predictor variable is an Exp(B) increase in the dependent variable (HAM-D score 12 months post scan), CI confidence interval, dlPFC dorsolateral prefrontal cortex.

**Table S8. A.** Working memory-related activity predicting Hamilton Depression scale scores 12 months post scan in the new sample after excluding individuals with an ADHD diagnosis at baseline. **B.** Emotional regulation-related activity predicting Hamilton Depression scale scores 12 months post scan in the new sample after excluding individuals with an ADHD diagnosis at baseline.

| **Parameter** | | ***B*** | **Hypothesis test** | | **95% Cl for Exp(*B*)** | | | |
| --- | --- | --- | --- | --- | --- | --- | --- | --- |
|  |  |  | **Wald *χ*2** | **Sig.** | **Exp(*B*)** | **Lower** | **Upper** |  |
| **A. Working Memory** | |  |  |  |  |  |  |  |
| Baseline depression | | 0.155 | 6.533 | 0.011 | 1.167 | 1.037 | 1.314 |  |
| dlPFC | left | 0.545 | 30.037 | <.001 | 1.724 | 1.419 | 2.095 |  |
| Precuneus | right | 0.133 | 1.807 | 0.179 | 1.142 | 0.941 | 1.386 |  |
| **B. Emotional Regulation** | | |  |  |  |  |  |  |
| Baseline depression | | 0.338 | 35.237 | <.001 | 1.401 | 1.254 | 1.567 |  |
| dlPFC | left | 0.687 | 22.144 | <.001 | 1.987 | 1.493 | 2.645 |  |

Exp(B) odds ratio or a 1-unit change in predictor variable is an Exp(B) increase in the dependent variable (HAM-D score 12 months post scan), CI confidence interval, dlPFC dorsolateral prefrontal cortex.

- No individuals were medicated the new sample, so subsequent sensitivity analyses could not be completed.

**Table S9. A.** Working memory-related activity predicting Hamilton Depression scale scores 12 months post scan in the combined sample after excluding individuals with an MDD diagnosis at baseline. **B.** Emotional regulation-related activity predicting Hamilton Depression scale scores 12 months post scan in the combined sample after excluding individuals with an MDD diagnosis at baseline.

| **Parameter** | | ***B*** | **Hypothesis test** | | **95% Cl for Exp(*B*)** | |  |
| --- | --- | --- | --- | --- | --- | --- | --- |
|  |  |  | **Wald *χ*2** | **Sig.** | **Exp(*B*)** | **Lower** | **Upper** |
| **A. Working Memory** | |  |  |  |  |  |  |
| Baseline depression | | 0.152 | 85.278 | <.001 | 1.164 | 1.127 | 1.202 |
| dlPFC | left | 0.293 | 12.625 | <.001 | 1.341 | 1.141 | 1.577 |
| Precuneus | right | 0.344 | 21.059 | <.001 | 1.411 | 1.218 | 1.634 |
| **B. Emotional Regulation** | | |  |  |  |  |  |
| Baseline depression | | 0.101 | 35.025 | <.001 | 1.106 | 1.070 | 1.143 |
| dlPFC | left | 1.270 | 94.383 | <.001 | 3.559 | 2.755 | 4.598 |

Exp(B) odds ratio or a 1-unit change in predictor variable is an Exp(B) increase in the dependent variable (HAM-D score 12 months post scan), CI confidence interval, dlPFC dorsolateral prefrontal cortex.

**Table S10. A.** Working memory-related activity predicting Hamilton Depression scale scores 12 months post scan in the combined sample after excluding individuals with an anxiety disorder diagnosis at baseline. **B.** Emotional regulation-related activity predicting Hamilton Depression scale scores 12 months post scan in the combined sample after excluding individuals with an anxiety disorder diagnosis at baseline.

| **Parameter** | | ***B*** | **Hypothesis test** | | **95% Cl for Exp(*B*)** | |  |
| --- | --- | --- | --- | --- | --- | --- | --- |
|  |  |  | **Wald *χ*2** | **Sig.** | **Exp(*B*)** | **Lower** | **Upper** |
| **A. Working Memory** | |  |  |  |  |  |  |
| Baseline depression | | 0.138 | 39.842 | <.001 | 1.148 | 1.100 | 1.198 |
| dlPFC | left | 0.300 | 10.976 | <.001 | 1.350 | 1.130 | 1.613 |
| Precuneus | right | 0.450 | 26.866 | <.001 | 1.568 | 1.323 | 1.858 |
| **B. Emotional Regulation** | | |  |  |  |  |  |
| Baseline depression | | 0.116 | 27.274 | <.001 | 1.123 | 1.075 | 1.173 |
| dlPFC | left | 1.292 | 81.067 | <.001 | 3.639 | 2.747 | 4.821 |

Exp(B) odds ratio or a 1-unit change in predictor variable is an Exp(B) increase in the dependent variable (HAM-D score 12 months post scan), CI confidence interval, dlPFC dorsolateral prefrontal cortex.

**Table S11. A.** Working memory-related activity predicting Hamilton Depression scale scores 12 months post scan in the combined sample after excluding individuals with an ADHD diagnosis at baseline. **B.** Emotional regulation-related activity predicting Hamilton Depression scale scores 12 months post scan in the combined sample after excluding individuals with an ADHD diagnosis at baseline.

| **Parameter** | | ***B*** | **Hypothesis test** | | **95% Cl for Exp(*B*)** | |  |
| --- | --- | --- | --- | --- | --- | --- | --- |
|  |  |  | **Wald *χ*2** | **Sig.** | **Exp(*B*)** | **Lower** | **Upper** |
| **A. Working Memory** | |  |  |  |  |  |  |
| Baseline depression | | 0.135 | 89.064 | <.001 | 1.144 | 1.113 | 1.177 |
| dlPFC | left | 0.339 | 15.108 | <.001 | 1.404 | 1.183 | 1.666 |
| Precuneus | right | 0.310 | 14.287 | <.001 | 1.363 | 1.161 | 1.600 |
| **B. Emotional Regulation** | | |  |  |  |  |  |
| Baseline depression | | 0.088 | 36.229 | <.001 | 1.092 | 1.061 | 1.124 |
| dlPFC | left | 1.284 | 98.159 | <.001 | 3.611 | 2.801 | 4.655 |

Exp(B) odds ratio or a 1-unit change in predictor variable is an Exp(B) increase in the dependent variable (HAM-D score 12 months post scan), CI confidence interval, dlPFC dorsolateral prefrontal cortex.

**Table S12. A.** Working memory-related activity predicting Hamilton Depression scale scores 12 months post scan in the combined sample after excluding medicated individuals at baseline. **B.** Emotional regulation-related activity predicting Hamilton Depression scale scores 12 months post scan in the combined sample after excluding medicated individuals at baseline.

| **Parameter** | | ***B*** | **Hypothesis test** | | **95% Cl for Exp(*B*)** | |  |
| --- | --- | --- | --- | --- | --- | --- | --- |
|  |  |  | **Wald *χ*2** | **Sig.** | **Exp(*B*)** | **Lower** | **Upper** |
| **A. Working Memory** | |  |  |  |  |  |  |
| Baseline depression | | 0.136 | 96.071 | <.001 | 1.145 | 1.115 | 1.177 |
| dlPFC | left | 0.347 | 22.499 | <.001 | 1.415 | 1.226 | 1.634 |
| Precuneus | right | 0.300 | 13.959 | <.001 | 1.350 | 1.153 | 1.580 |
| **B. Emotional Regulation** | | |  |  |  |  |  |
| Baseline depression | | 0.088 | 37.811 | <.001 | 1.091 | 1.061 | 1.122 |
| dlPFC | left | 1.292 | 102.874 | <.001 | 3.639 | 2.835 | 4.671 |

Exp(B) odds ratio or a 1-unit change in predictor variable is an Exp(B) increase in the dependent variable (HAM-D score 12 months post scan), CI confidence interval, dlPFC dorsolateral prefrontal cortex.

**Table S13. A.** Working memory-related activity predicting Hamilton Depression scale scores 12 months post scan in the new sample without covarying for baseline depression severity. **B.** Emotional regulation-related activity predicting Hamilton Depression scale scores 12 months post scan in the new sample without covarying for baseline depression severity.

| **Parameter** |  | ***B*** | **Hypothesis test** | | **95% Cl for Exp(*B*)** | | |
| --- | --- | --- | --- | --- | --- | --- | --- |
|  |  |  | **Wald *χ*2** | **Sig.** | **Exp(*B*)** | **Lower** | **Upper** |
| **A. Working Memory** | |  |  |  |  |  |  |
| dlPFC | left | 0.570 | 50.737 | <.001 | 1.769 | 1.512 | 2.069 |
| Precuneus | right | 0.285 | 13.514 | <.001 | 1.329 | 1.142 | 1.547 |
| **B. Emotional Regulation** | |  |  |  |  |  |  |
| dlPFC | left | 1.017 | 62.707 | <.001 | 2.765 | 2.15 | 3.557 |

Exp(B) odds ratio or a 1-unit change in predictor variable is an Exp(B) increase in the dependent variable (HAM-D score 12 months post scan), CI confidence interval, dlPFC dorsolateral prefrontal cortex.

**Table S14. A.** Working memory-related activity predicting Hamilton Depression scale scores 12 months post scan in the combined sample without covarying for baseline depression severity. **B.** Emotional regulation-related activity predicting Hamilton Depression scale scores 12 months post scan in the combined sample without covarying for baseline depression severity.

| **Parameter** |  | ***B*** | **Hypothesis test** | | **95% Cl for Exp(*B*)** | | |
| --- | --- | --- | --- | --- | --- | --- | --- |
|  |  |  | **Wald *χ*2** | **Sig.** | **Exp(*B*)** | **Lower** | **Upper** |
| **A. Working Memory** | |  |  |  |  |  |  |
| dlPFC | left | 0.522 | 51.625 | <.001 | 1.686 | 1.462 | 1.944 |
| Precuneus | right | 0.551 | 65.549 | <.001 | 1.735 | 1.519 | 1.983 |
| **B. Emotional Regulation** | |  |  |  |  |  |  |
| dlPFC | left | 1.879 | 295.837 | <.001 | 6.545 | 5.284 | 8.108 |

Exp(B) odds ratio or a 1-unit change in predictor variable is an Exp(B) increase in the dependent variable (HAM-D score 12 months post scan), CI confidence interval, dlPFC dorsolateral prefrontal cortex.

**Table S15. A.** Working memory-related activity predicting Young Mania Rating scale scores 12 months post scan in the new sample without covarying for baseline mania/hypomania severity. **B.** Emotional regulation-related activity predicting Young Mania Rating scale scores 12 months post scan in the new sample without covarying for baseline mania/hypomania severity.

| **Parameter** |  | ***B*** | **Hypothesis test** | | **95% Cl for Exp(*B*)** | | |
| --- | --- | --- | --- | --- | --- | --- | --- |
|  |  |  | **Wald *χ*2** | **Sig.** | **Exp(*B*)** | **Lower** | **Upper** |
| **A. Working Memory** | |  |  |  |  |  |  |
| dlPFC | left | -0.021 | 0.041 | 0.840 | 0.979 | 0.794 | 1.206 |
| Precuneus | right | 0.120 | 1.117 | 0.291 | 1.128 | 0.902 | 1.410 |
| **B. Emotional Regulation** | |  |  |  |  |  |  |
| dlPFC | left | 0.189 | 1.196 | 0.274 | 1.208 | 0.861 | 1.695 |

Exp(B) odds ratio or a 1-unit change in predictor variable is an Exp(B) increase in the dependent variable (HAM-D score 12 months post scan), CI confidence interval, dlPFC dorsolateral prefrontal cortex.

**Table S16. A.** Working memory-related activity predicting Young Mania Rating scale scores 12 months post scan in the combined sample without covarying for baseline mania/hypomania severity. **B.** Emotional regulation-related activity predicting Young Mania Rating scale scores 12 months post scan in the combined sample without covarying for baseline mania/hypomania severity.

| **Parameter** |  | ***B*** | **Hypothesis test** | | **95% Cl for Exp(*B*)** | | |
| --- | --- | --- | --- | --- | --- | --- | --- |
|  |  |  | **Wald *χ*2** | **Sig.** | **Exp(*B*)** | **Lower** | **Upper** |
| **A. Working Memory** | |  |  |  |  |  |  |
| dlPFC | left | 0.156 | 2.085 | 0.149 | 1.169 | 0.946 | 1.446 |
| Precuneus | right | 0.217 | 5.625 | 0.018 | 1.243 | 1.038 | 1.487 |
| **B. Emotional Regulation** | |  |  |  |  |  |  |
| dlPFC | left | 0.481 | 18.505 | <.001 | 1.618 | 1.299 | 2.014 |

Exp(B) odds ratio or a 1-unit change in predictor variable is an Exp(B) increase in the dependent variable (HAM-D score 12 months post scan), CI confidence interval, dlPFC dorsolateral prefrontal cortex.

**Figure S1.** Working memory (red)- and emotional regulation (blue)-related activity across the whole brain in the new sample. WM contrast is 2-backWM > 0-backWM. ER contrast is 2-backER > 0-backWM. Abbreviations: WM working memory; ER emotional regulation, dlPFC dorsolateral prefrontal cortex, pFWE family-wise error corrected, k cluster size.

**Discussion**

The ER models exhibited greater multivariate dissimilarity between the new and combined samples than the WM models. This finding likely reflects the fact that the ER component of the task showed greater interindividual variability in recruitment of underlying neural networks than the WM component of the task, due to the more complex nature of the ER than the WM component.

**References**

1.	Hamilton M. A rating scale for depression. J Neurol Neurosurg Psychiatry. 1960;23(1):56-62.

2.	Hamilton M. The assessment of anxiety states by rating. Br J Med Psychol. 1959;32(1):50-5.

3.	Young RC, Biggs JT, Ziegler VE, Meyer DA. A rating scale for mania: reliability, validity and sensitivity. Br J Psychiatry. 1978;133:429-35.

4.	Snaith RP, Hamilton M, Morley S, Humayan A, Hargreaves D, Trigwell P. A scale for the assessment of hedonic tone the Snaith-Hamilton Pleasure Scale. Br J Psychiatry. 1995;167(1):99-103.

5.	Overall JE, Gorham, D.R. The brief psychiatric rating scale. Psychol Rep. 1962;10:799-812.

6.	Hafkenscheid A. Psychometric evaluation of a standardized and expanded Brief Psychiatric Rating Scale. Acta Psychiatr Scand. 1991;84(3):294-300.

7.	Kessler RC, Andrews G, Colpe LJ, Hiripi E, Mroczek DK, Normand SL, et al. Short screening scales to monitor population prevalences and trends in non-specific psychological distress. Psychol Med. 2002;32(6):959-76.

8.	Watson D, Clark LA, Tellegen A. Development and validation of brief measures of positive and negative affect: the PANAS scales. J Pers Soc Psychol. 1988;54(6):1063-70.

9.	Hooper LM, Stockton, P. Krupnick, J.L., Green, B.L. Development, use, and psychometric properties of the Trauma History Questionnaire. J Loss Trauma. 2011;16:258-83.

10.	Clark LA, Watson D. Tripartite model of anxiety and depression: psychometric evidence and taxonomic implications. J Abnorm Psychol. 1991;100(3):316-36.

11.	Endicott J, Nee J, Harrison W, Blumenthal R. Quality of Life Enjoyment and Satisfaction Questionnaire: a new measure. Psychopharmacol Bull. 1993;29(2):321-6.

12.	Spielberger C. State-Trait Anxiety Inventory. The Corsini Encyclopedia of Psychology: Wiley; 2010.

13.	Cauffman E, Kimonis ER, Dmitrieva J, Monahan KC. A multimethod assessment of juvenile psychopathy: comparing the predictive utility of the PCL:YV, YPI, and NEO PRI. Psychol Assess. 2009;21(4):528-42.

14.	First MB, Williams, J. B. W., Karg, R. S., Spitzer, R. L. Structured Clinical Interview for DSM-5—Research Version (SCID-5 for DSM-5, Research Version; SCID-5-RV). American Psychiatric Association: Arlington, VA. 2015.

15.	Keller MB, Lavori PW, Friedman B, Nielsen E, Endicott J, McDonald-Scott P, et al. The Longitudinal Interval Follow-up Evaluation. A comprehensive method for assessing outcome in prospective longitudinal studies. Arch Gen Psychiatry. 1987;44(6):540-8.

16.	Blair JR, & Spreen, O. . Predicting premorbid IQ: A revision of the National Adult Reading Test. Clinical Neuropsychologist. 1989;3(2):129–36.

17.	Folstein MF, Folstein SE, McHugh PR. "Mini-mental state". A practical method for grading the cognitive state of patients for the clinician. J Psychiatr Res. 1975;12(3):189-98.

18.	Annett M. A classification of hand preference by association analysis. Br J Psychol. 1970;61:303-21.

19.	Leon AC, Solomon DA, Mueller TI, Turvey CL, Endicott J, Keller MB. The Range of Impaired Functioning Tool (LIFE-RIFT): a brief measure of functional impairment. Psychol Med. 1999;29(4):869-78.

20.	Patton JH, Stanford MS, Barratt ES. Factor structure of the Barratt impulsiveness scale. J Clin Psychol. 1995;51(6):768-74.

21.	Carver CS, White, T.L. Behavioral inhibition, behavioral activation, and affective responses to impending reward and punishment: the BIS/BAS scales. J Pers Soc Psychol. 1994;67.

22.	Cyders MA, Littlefield AK, Coffey S, Karyadi KA. Examination of a short English version of the UPPS-P Impulsive Behavior Scale. Addict Behav. 2014;39(9):1372-6.

23.	Ladouceur CD, Silk JS, Dahl RE, Ostapenko L, Kronhaus DM, Phillips ML. Fearful faces influence attentional control processes in anxious youth and adults. Emotion. 2009;9(6):855-64.

24.	Bertocci MA, Bebko GM, Mullin BC, Langenecker SA, Ladouceur CD, Almeida JR, et al. Abnormal anterior cingulate cortical activity during emotional n-back task performance distinguishes bipolar from unipolar depressed females. Psychol Med. 2012;42(7):1417-28.

25.	Bertocci MA, Afriyie-Agyemang Y, Rozovsky R, Iyengar S, Stiffler R, Aslam HA, et al. Altered patterns of central executive, default mode and salience network activity and connectivity are associated with current and future depression risk in two independent young adult samples. Mol Psychiatry. 2023;28(3):1046-56.

26.	Bertocci MA, Bebko G, Dwojak A, Iyengar S, Ladouceur CD, Fournier JC, et al. Longitudinal relationships among activity in attention redirection neural circuitry and symptom severity in youth. Biol Psychiatry Cogn Neurosci Neuroimaging. 2017;2(4):336-45.

27.	Fournier JC, Bertocci M, Ladouceur CD, Bonar L, Monk K, Abdul-Waalee H, et al. Neural function during emotion regulation and future depressive symptoms in youth at risk for affective disorders. Neuropsychopharmacology. 2021;46(7):1340-7.

28.	Afriyie-Agyemang Y, Bertocci MA, Iyengar S, Stiffler RS, Bonar LK, Aslam HA, et al. Lifetime depression and mania/hypomania risk predicted by neural markers in three independent young adult samples during working memory and emotional regulation. Mol Psychiatry. 2025;30(3):870-80.

29.	Esteban O, Markiewicz CJ, Blair RW, Moodie CA, Isik AI, Erramuzpe A, et al. fMRIPrep: a robust preprocessing pipeline for functional MRI. Nat Methods. 2019;16(1):111-6.

30.	Cox RW. AFNI: Software for Analysis and Visualization of Functional Magnetic Resonance Neuroimages. Comput Biomed Res. 1996;29:162-73.

31.	Kundu P, Voon V, Balchandani P, Lombardo MV, Poser BA, Bandettini PA. Multi-echo fMRI: A review of applications in fMRI denoising and analysis of BOLD signals. Neuroimage. 2017;154:59-80.

32.	Kundu P, Inati SJ, Evans JW, Luh WM, Bandettini PA. Differentiating BOLD and non-BOLD signals in fMRI time series using multi-echo EPI. Neuroimage. 2012;60(3):1759-70.

33.	DuPre E, Salo, T., Ahmed, Z., et al. TE-dependent analysis of multi-echo fMRI with *tedana*. J Open Source Software. 2021;6:3669.

34.	Smith SM, Jenkinson M, Woolrich MW, Beckmann CF, Behrens TE, Johansen-Berg H, et al. Advances in functional and structural MR image analysis and implementation as FSL. Neuroimage. 2004;23 Suppl 1:S208-19.

35.	Jenkinson M, Beckmann CF, Behrens TE, Woolrich MW, Smith SM. Fsl. Neuroimage. 2012;62(2):782-90.

36.	Gorgolewski K, Burns CD, Madison C, Clark D, Halchenko YO, Waskom ML, et al. Nipype: a flexible, lightweight and extensible neuroimaging data processing framework in python. Front Neuroinform. 2011;5:13.

37.	Friedman J, Hastie, T., Simon, N., Tibshirani, R. GLMNET. 2014.
